# *Taenia crassiceps* Infection Does Not Influence the Development of Experimental Rheumatoid Arthritis

**DOI:** 10.1155/2013/316980

**Published:** 2012-12-27

**Authors:** Aaxin M. Ortiz-Flores, Yadira Ledesma-Soto, Elsa A. Calleja, Miriam Rodríguez-Sosa, Imelda Juárez, Luis I. Terrazas

**Affiliations:** ^1^Unidad de Biomedicina, Facultad de Estudios Superiores Iztacala, Universidad Nacional Autónoma de México (UNAM), 54090 Tlalnepantla, MEX, Mexico; ^2^Carrera de Medicina, Facultad de Estudios Superiores Iztacala, Universidad Nacional Autónoma de México (UNAM), 54090 Tlalnepantla, MEX, Mexico

## Abstract

It was previously reported by our group that infection with *Taenia crassiceps* reduces incidence and severity of inflammatory and autoimmune experimental diseases like type 1 diabetes and experimental autoimmune encephalomyelitis. In this research, we set out to study whether infection with *T. crassiceps* would affect the development of experimental rheumatoid arthritis (RA). We found that mice infected with the parasite and induced with experimental RA showed similar clinical scores as the noninfected experimental RA group; systemic cytokines were not affected while anti-CII Abs were higher in the infected group. Histological evaluation showed damage in both infected and noninfected experimental RA-induced groups and although some surface molecules such as PDL-2 and MR which are associated with immunomodulatory mechanisms were upregulated in the infected and RA-induced group as compared to the noninfected RA group, they did not exert any changes in the outcome of experimental RA. Thus, we determined that infection with *T. crassiceps* does not influence the outcome of experimental RA.

## 1. Introduction

Rheumatoid arthritis (RA) is a chronic inflammatory disease with an estimate prevalence of 0.5%–1% around the world; it is characterized by inflammation and tenderness of the joints, which could lead to physical incapacity and even premature death due to complications. RA is an autoimmune disease, although the immunopathogenesis of RA is very complex to define due to the fact that a definitive cause of the disease has not yet been elucidated. Furthermore, the genetic background, exposure to environmental factors, and infectious agents contribute to the susceptibility, making it a multifactorial disease [1, 2]. 

Different signaling and effector pathways take place in the synovium in RA, and as a result a cascade of pathophysiological events leads to the progressive destruction of the joint. In normal conditions the synovium is acellular, and the membrane is mainly composed of macrophages and synovial fibroblast that surround all of the joints, but in RA the synovial membrane is infiltrated by macrophages, synovial fibroblasts, T cells, and B cells. Chronic inflammation and the hyperplastic synovial membrane lead to articular destruction. Macrophages and synovial fibroblast are important sources of proinflammatory cytokines like TNF-*α* and IL-1*β*; these cytokines are involved in various mechanisms that perpetuate the inflammatory milieu [3], as they up regulate NF*κ*B which in turns trigger the release of more pro-inflammatory cytokines and chemokines (e.g., IL-6, IL-8); in addition, these cytokines induce the expression of matrix degrading enzymes, the metalloproteinases (MMP) MMP1, MMP3, MMP8, MMP13, and MMP14 [4]. Furthermore TNF-*α* and IL-1*β* also upregulate (receptor activator of nuclear factor-*κ*B (RANK) ligand RANKL), which is necessary for the differentiation of osteoclasts; these cells are responsible for bone remodeling, but in RA the constant up regulation of RANKL leads to bone destruction. The involvement of T cells in RA has been very clear, since T cells have been found in RA patients synovium [5]; classically RA as well as other autoimmune diseases was classified as being one of a Th-1 phenotype based on the cytokine milieu found in experimental models of RA [2]. But with the recent discovery of IL-17 producing Th17 cells, new evidence seems to implicate even more this phenotype that may synergize with TNF-*α* and IL-1*β*, although we cannot rule out the participation of Th1 cells [6]. 

Most of the insight that have been gained in the development in the field of immunology have been made possible by the use of experimental murine models; the gold standard model for the induction of RA has been the collagen induced arthritis (CIA) model, that is based on the induction of type II collagen (CII) antibodies by immunization of DBA/1 mice with heterologous CII in Complete Freund's Adjuvant (CFA), with onset of the disease around day 40–50 postinoculation of the CII, resulting in infiltrating and hyperplasic synovium with erosion of bone and cartilage. Among other models of experimental RA, one that is able to break self-tolerance by inducing CII Abs production without the immunization of CII, is an experimental model of adoptive T cell transfer, in which DO11.10 CD4^+^ T cells (which specifically recognize a small ovalbumin peptide, OVA_323-339_) are skewed to a Th-1 phenotype by IL-12 and after culture are i.v. transferred followed by immunization of OVA in CFA and challenged with heat aggregated OVA (HAO). This experimental model is characterized by synovial infiltration and CII antibody production [7].

In the last two decades there has been a marked increase in inflammatory autoimmune diseases including multiple sclerosis (MS), type 1 diabetes (T1D), inflammatory bowel diseases (IBD: chron's disease and ulcerative colitis), rheumatoid arthritis (RA), as well as allergy, especially in developed countries. The observation that early and constant contact with pathogens reduced allergy was introduced by David Strachan, which led to the now known hygiene hypothesis, which was later modified to include autoimmune diseases [8]. The increase in hygiene, use of antibiotics, and less contact with infectious agents like helminthes and their products appear to be factors in the raise of allergic and autoimmune diseases [9]. Helminth parasites are known to modulate exacerbated immune responses; evidence of this comes from the observations that the incidence of allergy and autoimmune diseases is lower in underdeveloped countries where contact with helminths is constant [9]. 

The growing reports on experimental models of helminth-based therapy for relieving symptoms of inflammatory autoimmune diseases have showed promising results [10], recently our group has proven that infection with *Taenia crassiceps* reduced incidence and severity to T1D [11] and experimental autoimmune encephalomyelitis (EAE, a model for MS) [12], and protection was associated with the immune-regulatory mechanisms induced by the parasite. Thus, the focus of this research was to characterize the immune response induced by the infection with *T. crassiceps* on the outcome of experimental RA.

## 2. Material and Methods

### 2.1. Mice

Six-to seven-week-old male BALB/cAnN mice were purchased from Harlan Laboratories (Mexico) and were used as adoptive transfer recipients. Ten-to twelve-week-old male DO11.10 BALB/c TCR Tg mice that contain CD4^+^ T cells expressing a TCR that recognizes the chicken OVA_323-339_ peptide complexed with the MHC class II molecule I-A^d^ (detected by the clonotypic mAb KJ1.26) were obtained from The Jackson Laboratories (Bar Harbor, Maine USA); all animals were housed in a pathogen-free environment at the FES-Iztacala, U.N.A.M. animal facility in accordance with Institutional and National guidelines.

### 2.2. Parasites and Infection

 Metacestodes of *Taenia crassiceps* were harvested in sterile conditions from the peritoneal cavity of female BALB/c mice after 2–4 months of infection. The cysticerci were washed four times in sterile phosphate buffered saline 1X (PBS 1X; 0.15 M NaCL, 0.01 M sodium phosphate buffer, pH 7.2) and used for mouse infection. Mice were infected with an intraperitoneal (i.p.) injection of 20 small, nonbudding cysticerci of *T. crassiceps* suspended in 0.3 mL of sterile PBS 1X.

### 2.3. Induction of Experimental RA

 After DO11.10 BALB/c TCR Tg mice were humanly sacrificed, spleens were extracted and CD4^+^ T cells were purified by positive selection with anti-CD4^+^ mAbs bound by anti-IgG MACS beads (Militenyi Biotec, Auburn, CA), according to manufacturer's instructions. APCs were obtained from the peritoneal cavity and cultured for 2 h in supplemented RPMI medium, then washed twice with RPMI to discard nonadherent cells from the plates (Costar, MA. USA). Th1 cell differentiation was carried out culturing 2 × 10^5^/mL CD4^+^ T cells with 2 × 10^6^/mL APC, 0.3 *μ*M OVA_323–339_, and 5 ng/mL IL-12 (Peprotech Rocky Hill, NJ, USA) and 10 *μ*g/mL anti-IL-4 mAb. After 72 hours of culture, cells were washed and harvested for cell transfer. A total of 2 × 10^6^ DO11.10 CD4^+^ cells was injected i.v. via the tail vein into BALB/c recipient mice, and control mice received sterile PBS 1X only. One day after adoptive transfer, mice were immunized (subcutaneous, s.c.) with 100 *μ*g of OVA in CFA [7]. 

### 2.4. Assessment of Experimental RA

 Ten days after immunization with OVA in CFA, all animals were injected s.c. close to both rear ankle joints with 100 *μ*g of HAO. Mice were monitored for sign of arthritis, and disease was scored based on erythema and swelling in each paw on a scale of 0–3, giving a maximum score of 6 per mouse. Paw thickness was measured for seven days with a dial caliper (Mitutoyo, Japan).

### 2.5. Histology

Mice were sacrificed, and hind paws were removed and fixed in paraformaldehyde. Tissue was, then, processed and embedded in paraffin; 7 *μ*m sections were cut for histological analysis and stained with H&E.

### 2.6. Anticollagen Specific IgG1 and IgG2 Abs Detection

 Briefly, 96 well microplates (Nunc, Denmark) were coated with CII diluted in 0.1 M de NaHCO_3_ (pH 9.6) to a final concentration of 10 *μ*g/mL; 100 *μ*L of the solution was added to each well and stored overnight at 4°C, then plate were washed twice in PBS 1X 0.05% Tween 20 (USB Corporation, USA, washing buffer). Plates were blocked in PBS 1X in 1% BSA (blocking buffer) by adding 200 *μ*L of the solution to each well and incubated 1 h at 37°C, then washed three times in washing buffer. Serum samples were diluted in series starting at 1 : 50 in blocking buffer, and 100 *μ*L of the diluted samples was added to the microplate and incubated 1 h at 37°C, then washed four times in washing buffer. Peroxidase conjugated antimouse IgG1 and IgG2a (Zymed, USA) was diluted at a concentration of 1 : 1000 in blocking buffer, 100 *μ*L were added to each well, and they were incubated 45 minutes at 37°C. Microplates were washed four times in washing buffer, 100 *μ*L of hydrogen peroxide (3% H_2_O_2_) in 11 mL of 2,2′-azino-bis(3-ethilbenzthiazoline-6-sulphonic acid) was prepared, and 100 *μ*L of the solution was added as substrate to each well; OD values were measured at 405 nm using the Multiskan Ascent Thermo Lab Systems (AIE, USA) microplate reader.

### 2.7. Cytokine Detection

 Peripheral blood was collected from tail snips at day 2, 5, and 15 postchallenge with HAO. Serum cytokine IL-1*β*, IFN-*γ* and IL-4 levels were measured by sandwich ELISA using commercial kits purchased from PeproTech (Mexico) following manufacturer instructions. Briefly, captured antibody 2 *μ*g/mL was diluted in 10 mL of PBS 1X, 100 *μ*L was added to each well in 96 well Maxisorb microplate (Nunc, Denmark), and was incubated overnight at 4°C. Microplates were then washed twice with washing buffer, and plates were then blocked by adding 200 *μ*L of blocking buffer to each well and incubated for 2 h at 37°C. Plates were then washed three times in washing buffer. Recombinant mouse cytokines were used for standard curves starting at a concentration of 50 ng to 0.01 ng. Serum samples were diluted at a concentration of 1 : 5 in 100 *μ*L of blocking buffer, plates were incubated two hours at 37°C. Plates were washed four times in washing buffer, biotinylated antibody final concentration 1 *μ*g/mL was diluted in blocking buffer, and 100 *μ*L of the solution was added to each well, plates were incubated one hour at 37°C, then washed four times in washing buffer. Avidin (PeproTech Inc, USA) was diluted 1 : 4000 in blocking buffer; 100 *μ*L of the solution was added to each well, and the plates were incubated 30 minutes at 37°C and then washed five times in washing buffer. 100 *μ*L of the same substrate was used as above. OD values were measured at 405 nm using the Multiskan Ascent Thermo Lab Systems (AIE, USA) microplate reader.

### 2.8. Isolation of Peritoneal Macrophages

 After mice were sacrificed at the end of the experiment peritoneal exudates cells (PEC's) were obtained using 5 mL of ice cold sterile PBS 1X, and the red blood cells were lysed by resuspending the cells in Boyle's solution. Following two washes, the viable cells were counted by trypan blue exclusion with a Neubauer hemocytometer. PECs were adjusted 1 × 10^6^/mL in modified RPMI medium.

### 2.9. Antigen Specific Stimulation

 Spleens were removed 15 days post-HAO injection, and total cells were counted by trypan blue exclusion and adjusted to 3 × 10^6^ and then plated to a final concentration of 3 × 10^5^ cells/well. Then, cells were cultured with 50 *μ*g/mL of OVA peptide for 72 h, supernatants were then collected and stored until cytokine determination. 

### 2.10. Analysis of Cell Surface Markers in Macrophages

 The Fc receptors in peritoneal macrophages were blocked with anti-mouse CD16/CD32 (Biolegend, CA, USA) and then stained with an FITC-conjugated monoclonal antibody against F4/80, PE-conjugated antibodies against PD-L1 and PD-L2, and APC-conjugated antimannose receptor antibody (all obtained from Biolegend). The stained cells were analyzed on a FACs Calibur flow cytometer using Cell Quest software (Becton Dickinson).

## 3. Results

### 3.1. Th1 Polarization Was Induced in the DO11.10 CD4^+^ T Cells

**Figure 1:**
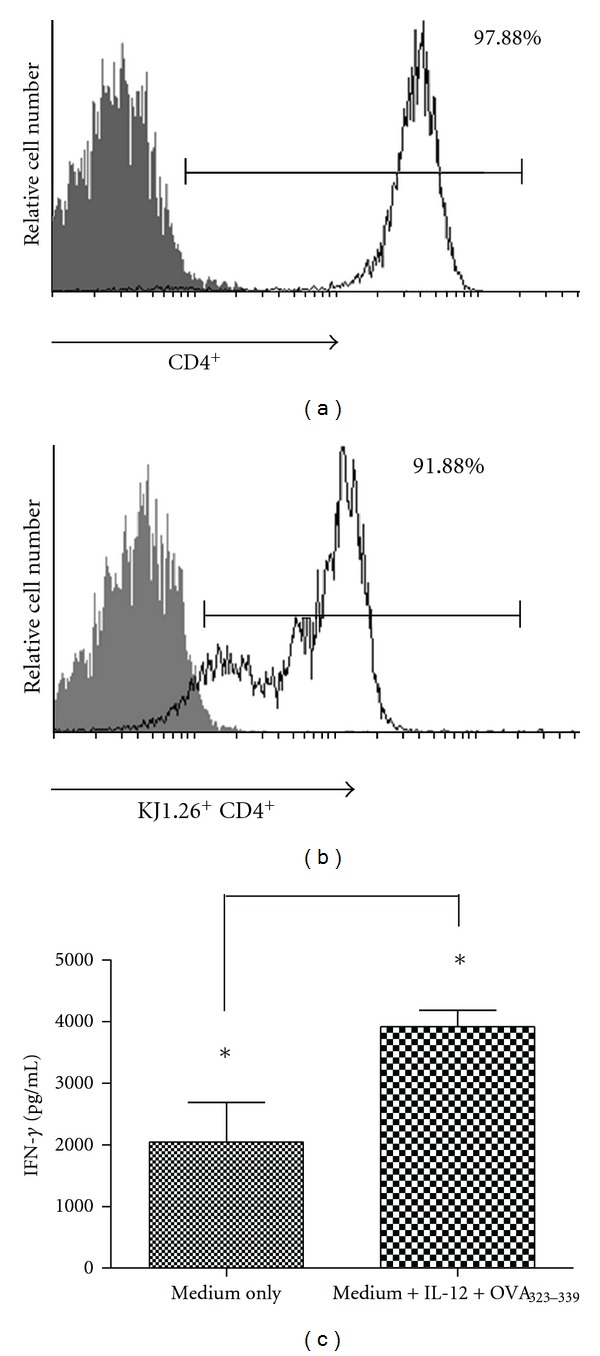
KJ1.26^+^ CD4^+^T cell purification and Th1 polarization of DO11.10 cells. CD4^+^ T cells from spleens of DO11.10 BALB/c TCR Tg mice were purified: (a) 97.88% were CD4^+^ and (b) 91.88% were KJ1.26^+^ CD4^+^. Th1 polarization, then, was induced in purified cells, (c) IFN-*γ* levels were measured in the supernatant of cocultures of DO11.10 CD4^+^ T cells, and macrophages, IL-12, and OVA_323-339_ peptide were used. *P < 0.05, *T*-test.

 CD4^+^ T cells from spleens of DO11.10 mice were purified by positive selection with anti-CD4^+^ mAbs (97.88% were CD4^+^, Figure 1(a)) and 91.88% were KJ1.26^+^ CD4^+^ (Figure 1(b)), and so more than 90% of the CD4^+^ cells were positive for the OVA TCR. Th1 polarization was then induced in these purified cells by coculture of DO11.10 CD4^+^ T cells and macrophages plus IL-12 and OVA_323-339_ peptide. IFN-*γ* levels were measured in the supernatants to verify the phenotype, and cells stimulated with OVA plus IL-12 produced significantly higher levels of IFN-*γ* as compared to the cells that received no stimuli (Figure 1(c)). Therefore, DO11.10 CD4^+^ T cells were polarized to a Th1 phenotype.

### 3.2. Assessment of Experimental Arthritis

**Figure 2:**
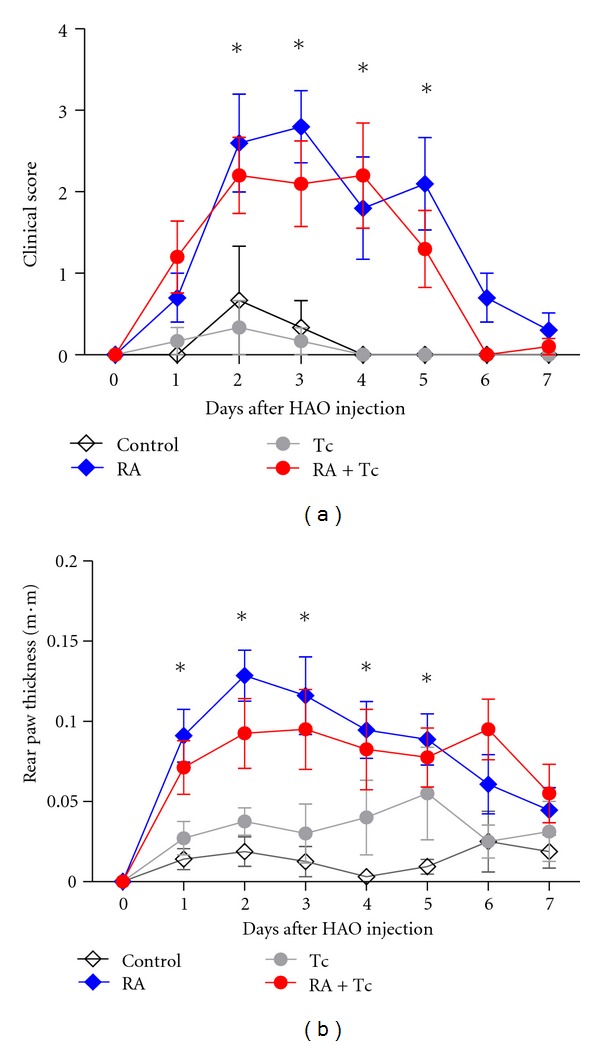
Clinical assessment of experimental arthritis. (a) Clinical score was evaluated based on erythema and swelling in each paw on a scale of 0–3, giving a maximum score of 6 per mouse. (b) Paw thickness was measured on both rear paws and was added to obtain an average measure for each mouse; the measures were taken seven days after HAO challenge. Statistical analysis, two-way ANOVA, *P < 0.05.

 Experimental RA was induced by the transfer of Th1-type DO11.10 CD4^+^ T cells in recipient BALB/c mice (RA), and after 6 weeks of infection with *T. crassiceps* (Tc + RA), control mice received unpolarized cells (Control), as well as the infected group (Tc); all groups were immunized with OVA in CFA followed by a periarticular injection near the ankle joints with HAO in both rear paws. RA was then evaluated in the four experimental groups based on erythema and swelling in each paw on a scale of 0–3, giving a maximum score of 6 per mouse for seven days. The not-transferred DO11.10 CD4^+^ T cell Control and Tc groups showed minimum clinical scores compared to the transferred RA and Tc + RA groups. The RA-induced group reached a peak in clinical signs around day 3 with a clinical score around 3 (Figure 2(a)); thereafter, clinical signs were reduced. Similarly, infected RA-induced mice (Tc + RA) reached a peak between days 2–4 with a maximum score of around 2. Tc+ RA group had slightly lower clinical scores than uninfected RA group, but differences were not significant among the two groups. Significant differences were found among the RA versus Control group on days 2–5, as well as with Tc + RA versus Tc group on days 2–4. Furthermore, measurements of paw thickness showed similar results within groups (Figure 2(b)), again the Control and Tc groups displayed minimum paw thickness. The RA group showed greater paw swelling than the Tc + RA group, which seem to have a slight tendency to decrease, but was not enough to show statistically significant differences between these groups. In contrast, the Control group showed significant differences with both transferred groups, on days 1–5 with the RA group and on days 2–4 with the Tc + RA group. These similar results obtained from the clinical scores and measurements of the paw thickness showed that infection with *T. crassiceps* does not seem to influence the outcome of experimental RA.

### 3.3. Histological Evaluation

**Figure 3:**
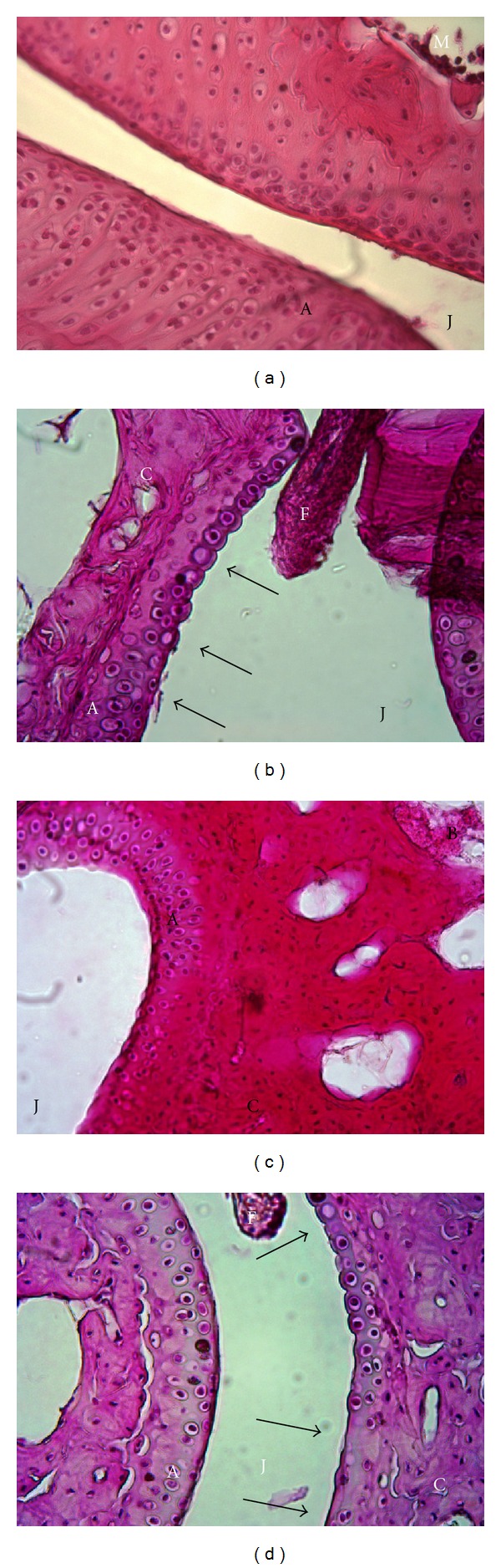
Histological evaluation. H and E staining was performed on sections of the distal phalanges joints. (a) Control; (b) RA; (c) Tc; (d) Tc + RA. Arrows show empty chondrocytes as well as isogenic bodies on cartilage. Magnification showed is 40X. J: joint cavity, A: articular cartilage, F: fibrous joint capsule, B: bone, C: calcified cartilage, and M: bone marrow. Arrows show empty chondrocytes as well as isogenic bodies on cartilage.

 After experimental RA was induced, 15 days post-HAO injection, all groups of mice were sacrificed; hind limbs were extracted, embedded in paraffin and sections of the distal phalanges joint were stained with H and E. The Control group (Figure 3(a)) and the Tc group (Figure 3(c)) showed normal arrangement of the joint cartilage, unlike the RA group (Figure 3(b)) which displayed empty chondrocytes which are characteristics of cartilage erosion; isogenic bodies were also found which are also commonly found in RA histology due to the fact that chondrocytes are in division trying to replace the loss of these cells in the cartilage of the joints; the same histological observations were seen in the Tc+RA group as well (Figure 3(d)). Since we found the same type of damage in the Tc + RA group as in the noninfected RA group, these results also indicated that infection with the parasite did not seem to modulate experimental RA.

### 3.4. Infected- and Experimental RA-Induced Mice Produce Higher Levels of Anti-CII IgG1 and IgG2a Antibodies

**Figure 4:**
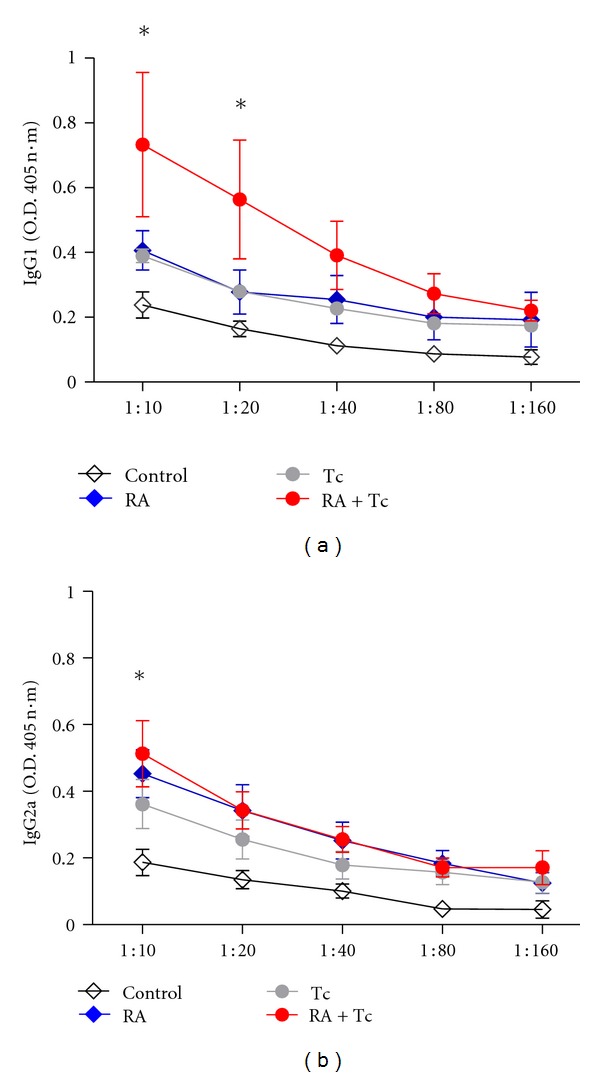
Infected and RA-induced mice produce higher levels of serum anti-CII IgG1 and IgG2 antibodies. Levels of (a) anti-CII IgG1; and (b) IgG2a Abs were measured in all of the groups sera were obtained 15 days post-HAO injection. Significant differences were observed in the IgG1 Abs between the RA versus Tc + RA and Tc versus Tc + RA in 1 : 10 dilution and Control versus Tc + RA in the 1 : 10 and 1 : 20 dilutions. In the IgG2a Abs Control versus RA and Control versus Tc + RA showed significant differences in the 1 : 10 dilution. Statistical analysis, two-way ANOVA, *P < 0.05.

 Anti-CII antibodies are pathogenic determinants of arthritis, therefore, IgG1 and IgG2a Abs were measured; the Control group produced irrelevant levels of anti-CII IgG1 and IgG2 Abs (Figures 4(a) and 4(b)). Interestingly, both infected groups Tc and Tc + RA produced higher levels of anti-CII IgG1 Abs even than the RA group. A similar finding was observed with anti-CII IgG2a. Labeling experiments have showed that many helminths possess a surface rich in collagen [13], so our data showing higher levels of anticollagen antibodies in the infected groups may be associated with this feature of the helminths surface. Significant differences were also observed in the IgG1 antibodies between the RA versus Tc + RA in the 1 : 10 dilution, whereas in the Control versus Tc + RA groups the difference was observed also in 1 : 20 dilution. In the IgG2a Abs Control versus RA and Control versus Tc + RA showed significant differences in the 1 : 10 dilution. These results are also in agreement with the data from the clinical score and measurements, since again infection with *T. crassiceps* did not reduce anti-CII IgG1 and IgG2a Abs, on the contrary both specific anti-CII antibodies were augmented. 

### 3.5. Infection with *T. crassiceps* Does Not Modify Systemic Serum Cytokine Levels

**Figure 5:**
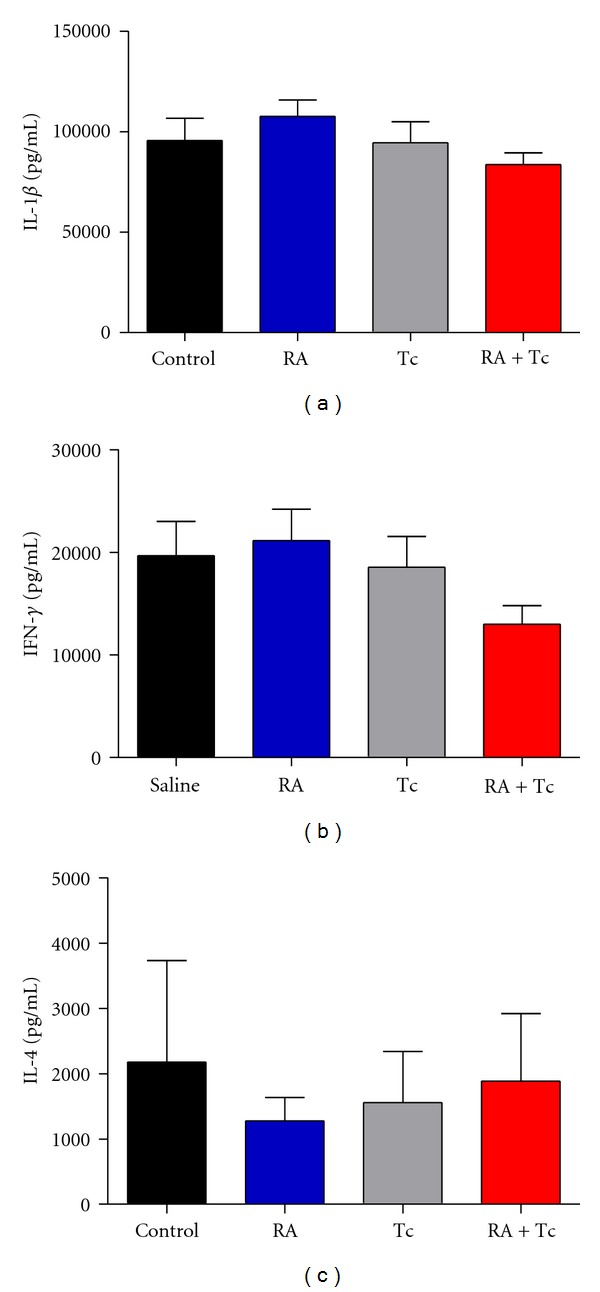
Infection with *T. crassiceps* does not modify systemic serum cytokine levels. Sera were obtained 5 days post-HAO injection, IL-1*β*, IFN-*γ*, and IL-4 cytokine levels were measured. No significant differences were observed within the groups.

Sera from all experimental groups were obtained 5 days post-HAO injection; Th1-type cytokines IL-1*β* (Figure 5(a)), IFN-*γ* (Figure 5(b)) and Th2-type cytokine IL-4 (Figure 5(c)) levels were measured. We did not observe significant differences within the groups. Although we did observe a tendency to decrease the levels in the proinflammatory cytokines IL-1*β* and IFN-*γ* in the infected groups and even more in the Tc + RA group, however, such differences did not reach significant values. Also the Tc + RA group displayed higher levels of IL-4 than Tc group; thus, this effect could be attributed to the parasite. The Control group had higher levels of IL-1*β* and IFN-*γ* than expected; this could have been the result of the strong Th1-type response induced by the immunization with OVA in CFA followed by the injection of the joints with HAO.

### 3.6. Antigen Specific IFN-*γ* Production

**Figure 6:**
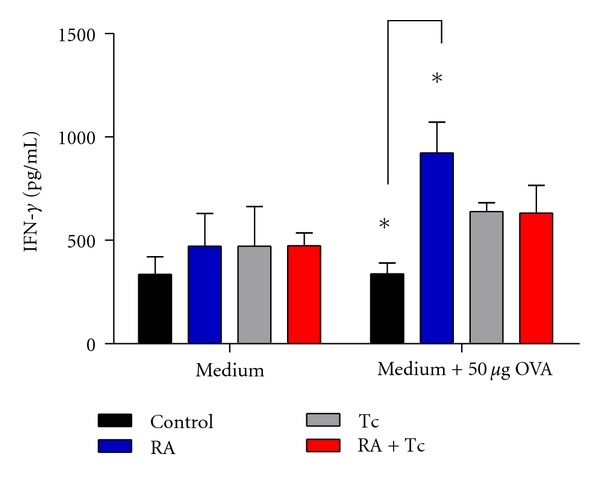
Antigen specific IFN-*γ* production. Spleens were extracted and cultured with medium alone or with OVA to determine IFN-*γ* levels. Significant differences were only observed in the Control versus RA group after stimulation. Statistical analysis, two-way ANOVA, *P < 0.05.

In order to determine if the antigen specific response was modified by the presence of *T. crassiceps* infection, total splenocytes were extracted and cultured with medium alone or stimulated with OVA to determine IFN-*γ* levels in the supernatants. Significant differences were observed in the Control versus RA group after stimulation with OVA, as expected after RA induction with OVA mice produced significant amounts of the Th1 cytokine (Figure 6). In agreement with previous works [14], *T. crassiceps* infection suppresses the antigen specific response of CD4^+^cells; thus, culture of splenocytes from *T. crassiceps*-infected groups did not produced significant amounts of IFN-*γ* after they were stimulated with OVA peptide.

### 3.7. AAM Surface Markers Are Upregulated in the RA-induced *T. Crassiceps*-Infected Mice

**Figure 7:**
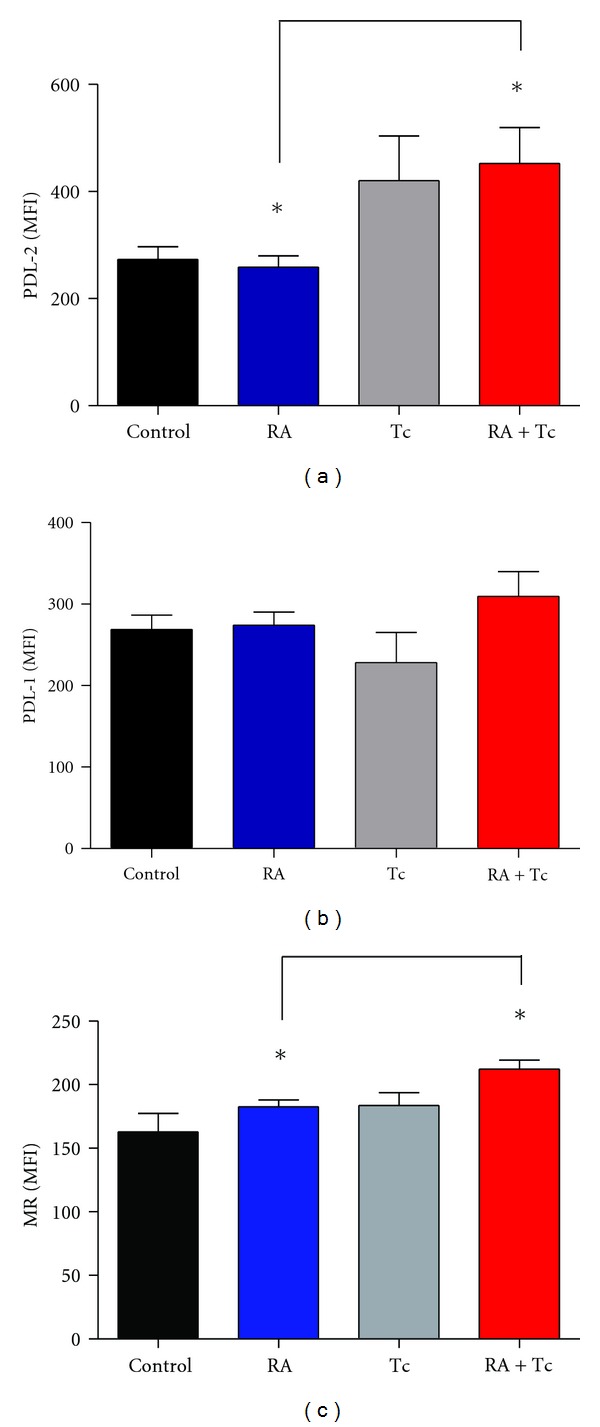
Flow cytometry analysis for alternatively activated macrophage (AAM) surface markers. AAM markers are up-regulated in the experimental rheumatoid arthritis induced on *T. crassiceps*-infected mice. Analysis of medium fluorescence intensity (MFI) on AAM markers PD-L1, PD-L2, and MMR was done. Statistical analysis, one-way ANOVA, *P < 0.05.

Flow cytometry was performed in order to analyze the presence of AAM during RA in mice-bearing *T. crassiceps*. MFI was analyzed on the AAM markers PD-L1 (Figure 7(a)), PD-L2 (Figure 7(b)), and MR (Figure 7(c)), and we found that the Tc + RA group had significantly increased expression of PDL-2 and MR on AAM than the noninfected groups; but in comparison with the Tc group, the differences were not statistically significant, although in both cases again it seems to be a pattern to increase in the Tc + RA group in contrast to the Tc group. AAM are known for their suppressive capacity; PD-ligands down-regulate T cell proliferation through direct contact with their receptor PD-1, usually expressed in T-activated cells [14]. The higher expression of PDL-2 and MR in Tc + RA could be an effect of the immunomodulatory mechanisms turned on for this parasite to down regulate the inflammatory response from the RA.

## 4. Discussion

In recent years there has been development of the now-called “helminth therapy” which basically states that infection with helminths or their products could potentially alleviate and improve inflammatory autoimmune diseases, due to the fact that helminths induce Th2 type phenotype that is able to antagonize the Th1 type phenotype of inflammatory autoimmune diseases as well as induce a series of regulatory cells such as AAMs, Tregs, and myeloid suppressor cells [10]. Proof of this comes from epidemiological observations, clinical studies as well as by experimental models and recent reviews [15].

Our group has recently demonstrated that infection with *T. crassiceps* reduces the incidence and severity to T1D [11], EAE [12], and colitis (unpublished observations) in murine models, and such protection has been associated with immunoregulatory mechanisms induced by this parasite. In contrast, the results showed by this study indicated that infection with *T. crassiceps* metacestodes does not alter the outcome of experimental RA.

Our data revealed that quantification of IgG2a anti-CII antibodies which are used as an indication of RA, showed, as expected, in the Contol group almost no production of anti-CII Abs, and the RA group did show significant amounts of antibodies in accordance with the disease, but unexpectedly the Tc + RA group had higher levels of IgG2a Abs compared to the RA group; apparently this could be due to the composition of the parasite itself [13] since the helminth own collagen could be interfering with the actual levels of CII antibodies due to the disease; this is an interesting fact that has not been reported before. Also the level of pro-inflammatory systemic cytokines showed similar results as the assessment of arthritis, where a tendency to decrease in the Tc + RA group was observed, but the other groups had similar amounts of IL-1*β* and IFN-*γ*; this could be due to the strong effects of the immunization with CFA plus OVA as well as the challenge injection with HAO. Infection with *T. crassiceps* increased expression of PDL-2 and MR which are markers of AAM in the Tc + RA compared to the RA non-infected group; it has been showed that infection with this parasite increased expression of PDL-1 and PDL-2 on AAM [14]; these ligands are involved in the PDL's pathway which is responsible for the inhibition of proliferative response of T cells in a contact-dependent manner. Thus, the increase of PDL-2 expression could be an attempt to inhibit the inflammatory response from arthritis, but, in this case the parasite immune modulation is just not enough to overcome the intense inflammatory response of the experimental RA. 

The gold standard model for the induction of experimental RA has been the collagen-induced arthritis (CIA) model, with onset of the disease around day 40–50 after inoculation of the CII, resulting in infiltrating and hyperplasic synovium, erosion of bone and cartilage, thus, displaying a very similar pathology as in humans [16]. Amelioration of CIA has been proven successful in infection with *Schistosoma mansoni* [17] and *S. japonicum* where is stage-dependent [18]; in both cases proinflammatory cytokines were down-regulated in infected mice as compared to noninfected. On the other hand, Osada and Kanazawa tried schistosome worm antigens (SWAP) or egg antigens (SEA), and either of them had effects on CIA [15]. Spontaneous arthritis is also another model used for studying this inflammatory disease; here it has been demonstrated that a gastrointestinal helminth such as *Nippostrongylus brasiliensis* [19] was able to down-modulate experimental arthritis. However, it is clear that one can expect that not all the helminth infections as well as the same helminth parasite would be able to cure or ameliorate every single inflammatory disease; in line with this idea, there has been occasions where infection with helminth parasites can turn out a whole range of possibilities as is the case in infection with *Hymenolepis diminuta*, where the infection with this parasite has augmented oxazolone-induced colitis in mice [20]; also this infection did not show any effects in acetic acid-induced ulceration in rats [21]; but, interestingly, infection with *H. diminuta* can be protected in dinitrobenzene sulfonic acid (DNBS)-induced colitis [22]. Therefore, even when infection is carried out with the same parasite, the outcome of the diseases can greatly vary, as in the case of *H. diminuta* even when in theory the same disease was being reproduced, although different methods of induction of the disease were used the results reflected an ample spectrum of possibilities. Moreover, another disease where any effect of a helminth infection was not detected was in EAE developed in mice carrying a gastrointestinal infection with *Strongyloides venezuelensis* [23]. Taken together all these data accumulated on helminth therapy, we suggest that more detailed studies are necessary before “generalize” that any single helminth parasite or their derivatives would be useful for any inflammatory or autoimmune disease. 

## 5. Conclusions

Based on the different outcomes of the many studies on “helminth therapy” and even though our group has previously proven that infection with *T. crassiceps* reduces incidence and severity of other inflammatory and autoimmune diseases, it is feasible that *T. crassiceps, *or any other helminth infection, does not affect the development of some disorders like experimental RA since most of inflammatory or autoimmune diseases have many different components involved that may be not affected by these parasites. Thus, in the context of the potential use of helminths or their molecules as a way to treat or improve the outcome of inflammatory or autoimmune diseases, it deserves larger and deeper studies before giving the title of a “close reality” to the use of such strategy.
